# Evaluation of the Physical and Shape Memory Properties of Fully Biodegradable Poly(lactic acid) (PLA)/Poly(butylene adipate terephthalate) (PBAT) Blends

**DOI:** 10.3390/polym15040881

**Published:** 2023-02-10

**Authors:** Marica Bianchi, Andrea Dorigato, Marco Morreale, Alessandro Pegoretti

**Affiliations:** 1 Department of Industrial Engineering and INSTM Research Unit, University of Trento, Via Sommarive 9, 38123 Trento, Italy; 2 Faculty of Engineering and Architecture, Kore University of Enna, Cittadella Universitaria, 94100 Enna, Italy

**Keywords:** PLA, PBAT, biodegradable polymers, blends, shape memory polymers

## Abstract

Biodegradable polymers have recently become popular; in particular, blends of poly(lactic acid) (PLA) and poly(butylene adipate terephthalate) (PBAT) have recently attracted significant attention due to their potential application in the packaging field. However, there is little information about the thermomechanical properties of these blends and especially the effect induced by the addition of PBAT on the shape memory properties of PLA. This work, therefore, aims at producing and investigating the microstructural, thermomechanical and shape memory properties of PLA/PBAT blends prepared by melt compounding. More specifically, PLA and PBAT were melt-blended in a wide range of relative concentrations (from 85/15 to 25/75 wt%). A microstructural investigation was carried out, evidencing the immiscibility and the low interfacial adhesion between the PLA and PBAT phases. The immiscibility was also confirmed by differential scanning calorimetry (DSC). A thermogravimetric analysis (TGA) revealed that the addition of PBAT slightly improved the thermal stability of PLA. The stiffness and strength of the blends decreased with the PBAT amount, while the elongation at break remained comparable to that of neat PLA up to a PBAT content of 45 wt%, while a significant increment in ductility was observed only for higher PBAT concentrations. The shape memory performance of PLA was impaired by the addition of PBAT, probably due to the low interfacial adhesion observed in the blends. These results constitute a basis for future research on these innovative biodegradable polymer blends, and their physical properties might be further enhanced by adding suitable compatibilizers.

## 1. Introduction

In recent years, the increasing difficulties of plastic disposal have raised concerns worldwide. Most plastic waste ends up in landfills, oceans, soil and water, representing thus serious hazards for plants, animals and humans. According to recent statistics, every year up to 12.7 million tons of plastic enter the oceans, causing the death of several seabirds and aquatic animals. The severity of this problem will exponentially increase with the increase in global non-biodegradable plastic consumption [1,2,3]. Plastic waste is estimated to triple by 2060, as claimed by the latest forecast by the OECD’s Global Plastic Outlook, rising from 353 million tons of waste in 2019 to 1014 million tons in the next decades [4]. Therefore, measures and solutions must be taken to avoid irreversible consequences on ecosystems and human beings. One of the major generators of plastic pollution is packaging, responsible for almost half of the global total due to its short lifespan [5]. This fact, highlighted in several statistics, has led to a growing demand for the use of biodegradable polymers for packaging materials since they could significantly minimize environmental pollution [5].

Nowadays, biodegradable polymers (BPs) have become a topic of great interest as an alternative to overcome the issues related to non-biodegradable polymeric waste. BPs can be decomposed by microbes (bacteria, some fungi and algae, etc.) existing in nature into CO_2_, H_2_O, CH_4_ and biomass, which can be integrated into the natural ecosystem without any ecotoxic effects [1]. One of the most studied BPs is poly(lactic acid) (PLA), a linear aliphatic polyester derived from lactic acid and synthesized from renewable resources, such as sugarcane or corn starch [6,7,8]. Due to its good mechanical properties (similar to those of polystyrene), processability, reasonable price and commercial availability, it has gained interest in academia and industry. PLA shows a Young’s modulus of around 3 GPa, a tensile strength between 50 and 70 MPa and an impact strength close to 2.5 kJ/m^2^ [9]. It can be processed at the industrial level with the same processing technologies used for traditional non-biodegradable polymers, but with lower greenhouse gas emissions [9]. PLA is the most used biodegradable polymer in the food packaging industry and it is commercialized mainly for single-use disposal packaging applications, such as bottles, cold drink cups, blister packages and flexible films [9]. It presents several advantages over non-biodegradable plastics that are commonly used for packaging, including good transparency, degradation in biological environments (for example, in soil and compost), biocompatibility and FDA approval [5]. In addition, it exhibits a thermo-responsive shape memory behavior that makes it even more attractive for the development of active and smart packaging [10,11,12,13,14,15,16].

In more detail, the polymer’s thermo-responsive shape memory behavior allows it to undergo large controllable shape changes in response to a change in temperature [17,18,19]. In the case of semi-crystalline PLA, the crystalline domains act as the net points and determine the initial permanent shape of the material, while the glass transition temperature of PLA (approx. 60 °C) acts as a molecular switch and is responsible for the fixability of the desired temporary shape. PLA macromolecules exhibit high mobility when the material is heated above its glass transition, allowing the polymer to be easily deformed into the appropriate temporary shape. Cooling PLA under loads below T_g_ strongly reduces its mobility, thereby fixing the imposed deformation. Finally, when the polymer is heated above T_g_, the switching domains progressively regain their mobility, and the material returns to its original condition [17,18,19].

Although PLA possesses many advantages for packaging, it also has some drawbacks that prevent its industrial exploitation, including poor toughness and sensitivity to thermal degradation [20,21]. One possible strategy to find more PLA commercial applications in the sustainable packaging field, while maintaining its biodegradability, consists of blending PLA with other BPs that have complementary characteristics [21]. Melt-blending techniques are generating a lot of interest since they are a cost-effective, easy and readily available processing technology at the industrial level that allows us to obtain packaging formulations with a desired performance by varying the blend composition [21]. Poly(butylene adipate-co-terephthalate) (PBAT), an aliphatic-aromatic copolymer, is considered an interesting toughening candidate [22]. It is a fully biodegradable polymer based on fossil resources, produced by poly-condensation between butanediol (BDO), adipic acid (AA) and terephthalic acid (PTA) [23,24]. PBAT is characterized by high elasticity, good thermal stability at elevated temperatures, wear and fracture resistance, and compatibility with many other natural polymers. However, its stiffness and strength are rather poor [22,23,24,25]. PBAT’s properties can be slightly adjusted by varying the relative ratio of the two structural units: the rigid butylene terephthalate (BT) segments, derived from 1,4-butanediol and terephthalic acid monomers, and the flexible butylene adipate (BA) segments, derived from 1,4-butanediol and adipic acid monomers. Similar to PLA, PBAT has found application in packaging (for example, in the production of plastic bags), as well as in the biomedical field, in hygiene products and in mulch film production [23,24,25].

In view of their complementary characteristics and common application fields, blending stiff PLA with flexible PBAT could represent a valuable technical solution to develop eco-sustainable packaging with tailorable properties [26,27,28]. PLA/PBAT blends have been thoroughly researched over the years [29,30,31,32,33,34,35,36,37]. Farsetti et al. [37] found that PLA/PBAT blends with different relative concentrations produced by melt compounding exhibited two-phase behavior and two separate glass transition temperatures. The blends maintained quite a high modulus and tensile strength compared to those of neat PLA, and small amounts of PBAT improved both the elongation at break and the impact resistance. Gu et al. [32] investigated the melt rheology of PLA/PBAT blends with PBAT contents lower than 30 wt% prepared by twin-screw extrusion. The incorporation of PBAT was found to improve the melt processability, since the PLA/PBAT blends were characterized by a more pronounced shear-thinning behavior compared to that of the neat PLA. Several studies have focused on the preparation and characterization of flexible PLA/PBAT biodegradable films for agricultural uses. For example, Weng et al. [33] prepared PLA/PBAT films and investigated their biodegradation behavior in a real soil environment. They observed that the blends presented a lower biodegradation rate compared to that of the neat polymers, and this experimental evidence was attributed to the poor compatibility between the PLA and PBAT phases, as highlighted by SEM micrographs. Palsikowski et al. [36] prepared flexible PBAT/PLA films compatibilized with a chain extender and investigated the effect of the addition of the chain extender on their biodegradation. The result highlighted that the presence of the chain extender led to a delay in the film biodegradation, and the blends showed an intermediate behavior compared to that of the neat polymers. However, few studies in the literature have investigated the use of these blends in the packaging field, and the majority of these studies have primarily dealt with food packaging. For example, Paulsen et al. [26] studied the suitability of PLA/PBAT films in the packaging of broccoli. They found that broccoli heads packaged in these films achieved a shelf life of 21 days at 2 °C, which is a more extended postharvest storage with respect to that of low-density polyethylene films. Weng et al. [27] discovered that cinnamaldehyde-loaded corn starch/PBAT/PLA blend film effectively preserved the quality of soy-protein-based meat analogues by inhibiting the growth of *Escherichia coli* and *Staphylococcus aureus* during 4 °C storage. Qui et al. [28] produced PLA/PBAT films incorporated with nano-polyhedral oligomeric silsesquioxane (POSS (epoxy)8) as a reactive compatibilizer via melt processing. They noticed that the addition of POSS to the PLA/PBAT films improved the storage capacity of the packaging films and that the excellent permeability of PBAT created a low-moisture environment capable of suppressing fungal growth.

Considering the recent interest in PLA/PBAT blends and in the shape memory capability of PLA for the development of smart packaging with a thermo-responsive shape, this work is focused on the investigation of the thermomechanical and shape memory properties of fully biodegradable PLA/PBAT blends, produced by melt compounding and hot pressing. Therefore, this study aims at investigating the effect of the blend composition on the processability and on the most important physical features of these materials, in view of their future application in the packaging field. To the authors’ knowledge, this is the first work dealing with the investigation of the shape memory behavior of PLA/PBAT blends.

## 2. Materials and Methods

### 2.1. Materials and Methods

Ingeo^TM^ 2500HP, supplied by NatureWorks^®^ LLC (Minnetonka, MN, USA) in pellet form, was the PLA used in this work. According to the producer’s technical datasheet, it was characterized by a specific gravity of 1.24 g/cm^3^, a melt flow index (MFI) of 8 g/10 min (210 °C, 2.16 kg) and a melting temperature of 165–180 °C. Polymer granules of PBAT Technipol^®^ Bio 1160 were purchased from Sipol Spa (Mortara, PV, Italy). According to the supplier datasheet, its specific gravity was 1.23 g/cm^3^, the MFI was 20 g/10 min (160 °C, 2.16 kg) and melting temperature was about 115 °C.

### 2.2. Sample Preparation

PLA/PBAT blends at different relative ratios were prepared by melt compounding in a Rheomix 600 internal mixer (Thermo Haake^®^, Waltham, MA, USA), equipped with counter-rotating rotors. The compounding temperature was kept at 180 °C while the mixing time was 10 min. The rotor speed was set at 60 rpm. Prior to processing, PLA and PBAT pellets were dried at 50 °C overnight to remove moisture. After compounding, the blends were hot-pressed in a hydraulic press for 10 min at a pressure of 7 bar and a temperature of 180 °C, followed by water cooling. The processing temperature was selected after preliminary trials, and viscosimetric measurements on both virgin and processed samples (not reported for the sake of brevity) demonstrated that the molecular weight of the constituents was retained after melt-compounding and hot-pressing operations. In this way, square sheets with in-plane dimensions of 150 × 150 mm^2^ and a thickness of 1 mm were prepared. The composition (in wt% and vv%) of the prepared samples along with their codes is reported in Table 1. The final number in the code refers to the PBAT weight concentration in the blend.

### 2.3. Characterization

#### 2.3.1. Rheological Properties

To examine how the blend’s composition affects the viscosity in the molten state, dynamic rheological tests were performed on a DHR-2 rheometer (TA Instrument, New Castle, DE, USA) in parallel plate oscillatory mode. The measurements were conducted using 25 mm diameter plates at 180 °C and setting a gap distance of 1 mm. Disc samples (diameter 25 mm, thickness 1 mm) were selected for the tests. The measurements were conducted in frequency sweep mode, applying a maximum shear strain of 1%, which, according to the literature, is within the limit of viscoelasticity for these systems [38]. In this way, the trends of the complex viscosity (η*), storage modulus (G′) and loss modulus (G″) were evaluated in an angular frequency range of 0.1–1000 rad/s.

#### 2.3.2. Microstructural Properties

In order to investigate the dispersion of the two constituents in the blends, optical microscope micrographs were acquired on polished samples, by using an upright incident-light optical CH-9435 Heerbrugg microscope (Heerbrugg, Switzerland).

A field emission scanning electron microscope (FESEM) AG-SUPRA 40 (Carl Zeiss, Ober-Kochen, Germany), operating at an accelerating voltage of 2.5 kV, was employed to investigate the interfacial adhesion between the PLA and PBAT phases. The samples were cryofractured after being submerged in liquid nitrogen for one hour. The fracture surface was analyzed after Pd/Pt sputtering to provide enhanced electrical conductivity.

#### 2.3.3. Thermal Properties

In order to investigate the degradation resistance of the produced blends, thermogravimetric analysis (TGA) was performed by using a Mettler TG50 thermo-balance (Mettler-Toledo GmbH, Schwerzenbach, Switzerland). Samples with a mass of 20 mg were tested at a heating rate of 10 °C/min from 25 °C up to 700 °C under a nitrogen flow of 10 mL/min. The temperature that was associated with a mass loss of 5% (T_5%_) and the residual mass at 700 °C (m_700_) were evaluated. In addition, the temperatures corresponding to the maximum mass loss rate of PLA (T_peak1_) and PBAT (T_peak2_) were determined from the first derivative of the thermogravimetric curve (DTG).

Differential scanning calorimetry (DSC) tests were performed on the prepared blends by means of a Mettler DSC30 calorimeter (Mettler Toledo GmbH, Schwerzenbach, Switzerland). Measurements were carried out under 100 mL/min nitrogen flow according to the following protocol: (I) the first heating scan was from −80 °C to 200 °C at 10 °C/min, (II) the cooling scan was from 200 °C to −80 °C at 10 °C/min, (III) and the final heating scan was from −80 °C to 200 °C at 10 °C/min. Specimens with a mass of 10 mg were tested. DSC analysis allowed the determination of the glass transition temperature (T_g_), the melting and cold crystallization temperatures (T_m_, T_cc_) and specific enthalpy values ($\Delta H_{m},\;\Delta H_{cc}$) of the blend constituents. Furthermore, the PLA and PBAT crystallinity degree (χ_c_) was calculated according to Equation (1):

(1) $$\chi_{c\;}\left(\%\right)=100\cdot \frac{\Delta H_{m}-\Delta H_{cc}}{\Delta H_{m}^{0}.w}$$

where $\Delta H_{cc}$ is the cold crystallization enthalpy, $\Delta H_{m}^{0}$ is the theoretical melting enthalpy of fully crystalline polymer and *w* is the weight fraction of the polymer phase in the blend. $\Delta H_{m}^{0}$ was taken as 93 J/g for fully crystalline PLA and 114 J/g for fully crystalline PBAT [20,24].

#### 2.3.4. Mechanical Properties

Dynamic-mechanical analysis was carried out through a DMA Q800 (TA instrument, New Castle, DE, USA) machine, in order to investigate the dependence of the storage modulus (E’) and loss modulus (E”) of the produced blends on temperature. The tests were performed on rectangular specimens (gauge length 10 mm, width 5 mm, thickness 1 mm) with a frequency of 1 Hz, a strain amplitude of 0.05% and a heating ramp from 25 °C to 130 °C at 3 °C/min. One specimen was tested for each composition. In this way, the values of the storage modulus at 25 °C (E’_25_) and at 85 °C (E’_85_) were determined to evaluate the storage modulus drop below and above the glass transition temperature of PLA.

An Instron 5969 (Instron^®^, Norwood, MA, USA) tensile testing machine, equipped with a load cell of 10 kN and operating at a cross-head speed of 5 mm/min, was used to determine the tensile properties of the blends under quasi-static conditions. The tests were performed on ISO 527 type 1BA specimens at 25 °C. The elastic modulus (E) was measured as the secant value between two strain levels (0.05 and 0.25 mm/mm) by means of a resistance extensometer. Moreover, the tensile stress at break (σ_b_) and the strain at break (ε_b_) were determined. Five specimens were tested for each composition.

#### 2.3.5. Evaluation of the Shape Memory Behavior

**Figure 1:**
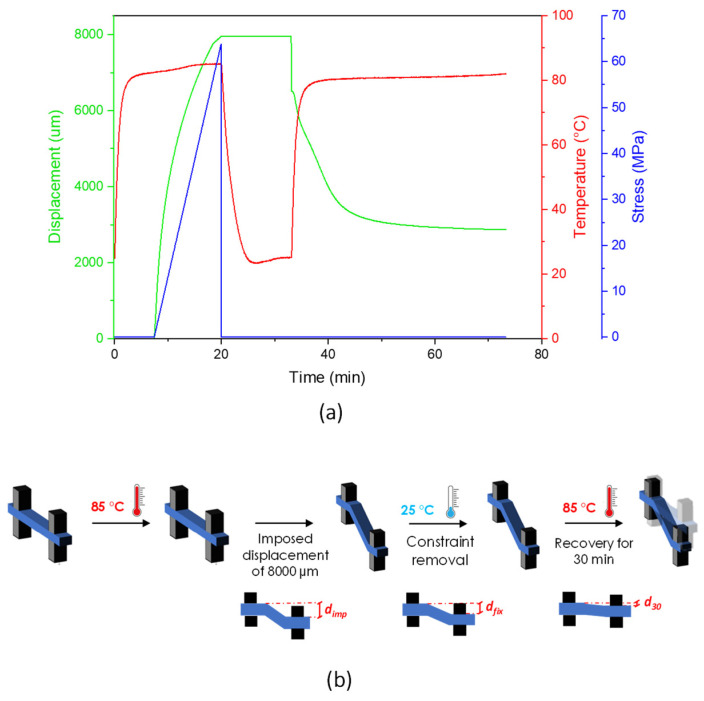
(**a**) Graphical representation, as an example, of PLA_PBAT_15 sample and (**b**) illustration of the thermomechanical cycle adopted to investigate the shape memory behavior of the PLA/PBAT blends.

The investigation of the shape memory behavior of the prepared PLA/PBAT blends was performed on prismatic rectangular specimens (width 5 mm, thickness 1 mm, length 20 mm) by means of a DMA Q800 (TA instrument, New Castle, DE, USA) apparatus, operating in single cantilever mode. Since at 25 °C and 85 °C the PLA phases in the blends are in the glassy and in the rubbery state, respectively, these temperatures were selected for exploring the shape memory performance of the samples. One specimen was tested for each formulation. The tests were carried out according to the thermomechanical cycle typically employed for SMPs [39,40,41,42,43,44,45,46,47,48], consisting of a programming step, followed by a recovery step. More specifically, the specimens were subjected to the following thermomechanical history: (I) heating of the specimen and equilibrium at 85 °C; (II) application of a ramp force of 0.8 N/min (the ramp force was reduced to 0.1 N/min for PLA_PBAT_75 blend); (III) drive-off of the motor when a displacement (d_imp_) of 8000 µm was achieved; (IV) cooling and equilibrium of the sample at 25 °C (cooling rate of 20 °C/min); (V) isothermal step for 5 min; (VI) drive-on of the motor and application of a constant load equal to 0.001 N to continuously monitor the sample displacement as a function of the temperature; (VII) equilibrium at 85 °C; and (VIII) isothermal for 30 min at 85 °C. The graphical representation and the schematization of the thermomechanical cycle is illustrated in Figure 1a,b (the graphical representation of the thermomechanical cycles of all blends are reported in Figure S1). In Figure 1b, d_imp_ is the programmed displacement at step (III), d_fix_ is the effectively fixed displacement at step (VI) and d_30_ is the displacement reached by the movable clamp at the end of the recovery process at step (VIII).

The strain fixity (SF) and strain recovery (SR) parameters were then determined in order to describe the shape memory performance of the PLA/PBAT blends. The first parameter assesses the material’s capacity to fix the mechanical deformation introduced during the programming process, while the second one indicates how much strain is recovered during the recovery step. Both SF and SR should be close to 100% in a well-performing SMP. For each specimen, the SF and SR were, respectively, calculated according to Equations (2) and (3):

(2) $$SF\left(\%\right)=\frac{d_{fix}}{d_{imp}}\cdot 100$$

(3) $$SR\left(\%\right)=\frac{d_{imp}-d_{30}}{d_{imp}}\cdot 100$$

## 3. Results and Discussion

### 3.1. Rheological Measurements

**Figure 2:**
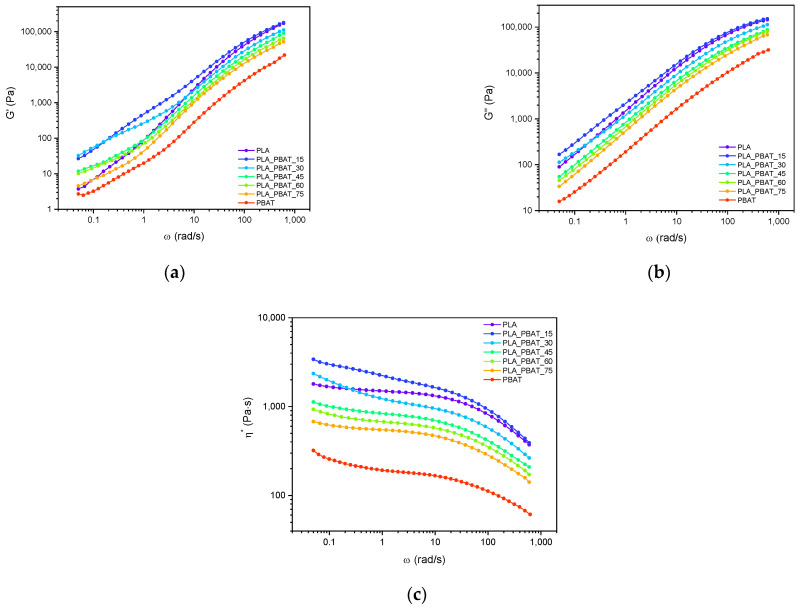
Results of the dynamic rheological tests at 180 °C on the neat matrices and on the prepared blends. Trends of (**a**) storage modulus; (**b**) loss modulus; and (**c**) complex viscosity as a function of the angular frequency.

Dynamic rheological tests were carried out to investigate the effect of the blend composition on the rheological properties at the processing temperature (180 °C) in order to obtain information about the processability of these materials. Figure 2a–c show the results of the dynamic rheological tests performed on neat PLA, neat PBAT and all the prepared blends in terms of the dynamic moduli (G′, G″) and complex viscosity (η*).

Figure 2a,b show, respectively, the storage and loss modulus of the PLA/PBAT blends as a function of the frequency. All the samples exhibit a similar trend of the G′ in the high-frequency range, while differences can be seen at lower frequencies. The G′ of all blends in the low-frequency range was higher than that of neat PLA and neat PBAT, suggesting that the two polymers are probably immiscible [49]. Furthermore, significant differences in the slopes of the curves appear at low frequencies, i.e., with the appearance of a shoulder in the PLA_PBAT_15 blend. This behavior is very likely to be due to a droplet/matrix morphology [50,51,52,53] and it substantially disappears at higher compositions. This could be attributed to the formation of more complex morphologies showing slow relaxation dynamics [51,52,53] and will be further investigated and discussed in the following morphological characterization section.

Comparing the loss and the storage modulus values, it can be observed that the G″ is greater than the G′; this, combined with their overall trends, suggests that a liquid-like rheological behavior prevails over a solid-like one for all of the investigated blends. Furthermore, the frequency dependence of the G″ is almost the same for all the samples in the entire frequency range. Finally, when increasing the PBAT content in the blends, both the G′ and G″ decrease.

The complex viscosity of the neat PLA and PBAT (Figure 2c) is similar to the values reported in the literature, highlighting the negligible thermal degradation these materials undergo during the production process [54]. On a general level, all the samples exhibited a shear thinning behavior, more pronounced in the high-frequency range. The η* of PLA is approximately one order of magnitude higher than that of PBAT, as shown in Figure 2c. Therefore, this suggests that it is much easier for PBAT to disperse within the PLA matrix, once processed at the molten state. With the increasing PBAT concentration, the complex viscosity of the blends decreases, except for that of PLA_PBAT_15 and PLA_PBAT_30 in the low-frequency regime, attributable to yield stress, likely to be due to resistance to the flow of the PBAT domains in the PLA matrix.

### 3.2. Morphological Characterization

**Figure 3:**
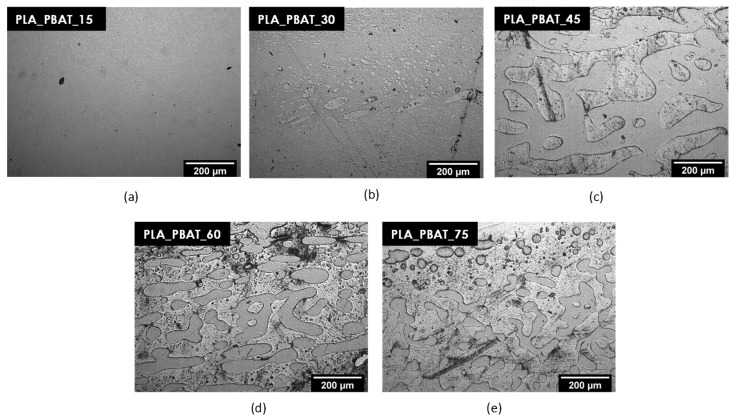
Optical microscope images of the prepared PLA/PBAT blends. (**a**) PLA_PBAT_15; (**b**) PLA_PBAT_30; (**c**) PLA_PBAT_45; (**d**) PLA_PBAT_60; and (**e**) PLA_PBAT_75.

The optical microscope images of the prepared blends are shown in Figure 3a–e.

The bright field micrographs shown in Figure 3a–e provide interesting information about the microstructural features of the produced PLA/PBAT blends. Firstly, it is possible to notice that PLA and PBAT are immiscible since all the analyzed compositions show an evident phase separation, which is consistent with the observation reported in other studies on these systems [22,35]. Secondly, depending on the PLA/PBAT ratio, the two phases exhibit either a sea-island morphology (Figure 3a,b,e), i.e., a discrete domain (regularly or irregularly shaped) embedded in the surrounding matrix, or an almost co-continuous structure (Figure 3c,d), i.e., large interconnected domains with irregular shapes. Focusing at first on blends with low PBAT contents, PLA_PBAT_15 (Figure 3a) presents a fine structure with small and spherical PBAT domains evenly dispersed in the PLA matrix. After a statistical analysis of the domain size distribution, it is possible to conclude that the size of PBAT domains increases with the PBAT concentration, from 3.6 ± 0.7 µm for the PLA_PBAT_15 blend up to 11.9 ± 8.9 µm for the PLA_PBAT_30 sample. Moreover, in the PLA_PBAT_30 blend (Figure 3b), there is the coexistence of small and large PBAT droplets, with either spherical or irregularly elongated shapes. The formation of these small PBAT domains in the PLA matrix could be responsible for the high complex viscosity values evidenced in the low-frequency range in the PLA_PBAT_15 and PLA_PBAT_30 samples (see Figure 2c). The dispersed PBAT phase probably hindered the mobility of the PLA chains, inducing a viscosity increase. A significant change in morphology is observable in the PLA_PBAT_45 blend (Figure 3c). This sample exhibits an almost co-continuous morphology, with a 3D network of large and elongated PBAT domains in the PLA matrix. Moreover, it is interesting to observe that small PLA droplets are immersed in some PBAT domains. This experimental evidence suggests that PLA/PBAT is a polymer blend characterized by high viscosity, since double-emulsion structures are generally present in blends with this feature [55]. As can be seen in Figure 3d, PLA_PBAT_60 also presents a co-continuous morphology. However, differently to PLA_PBAT_45, PBAT is the primary constituent in this case, highlighting that in the PBAT concentration interval between 45 wt% and 60 wt%, the phase inversion occurred. Future works will aim at identifying the exact composition associated to this inversion. By a more detailed analysis of the blend containing 60 wt% of PBAT, it is possible to observe that PLA domain size and shape are broadly distributed, since small PLA droplets are present in the PBAT matrix together with the coarser domains. Finally, by further increasing the PBAT content in the blends up to 75 wt% (Figure 3e), the size of the PLA domains, as well as the statistical dispersion of their size distribution, decreases. Their elongated shape probably derives from the coalescence of smaller domains. In conclusion, it is interesting to point out that, on a general level, when PBAT is in the dispersed phase of the blend, its domains are smaller than those formed by PLA when PBAT is the primary constituent. In fact, as it has been evidenced by rheological measurements, PBAT presents a significantly lower viscosity, which favors its breakup in the PLA matrix.

**Figure 4:**
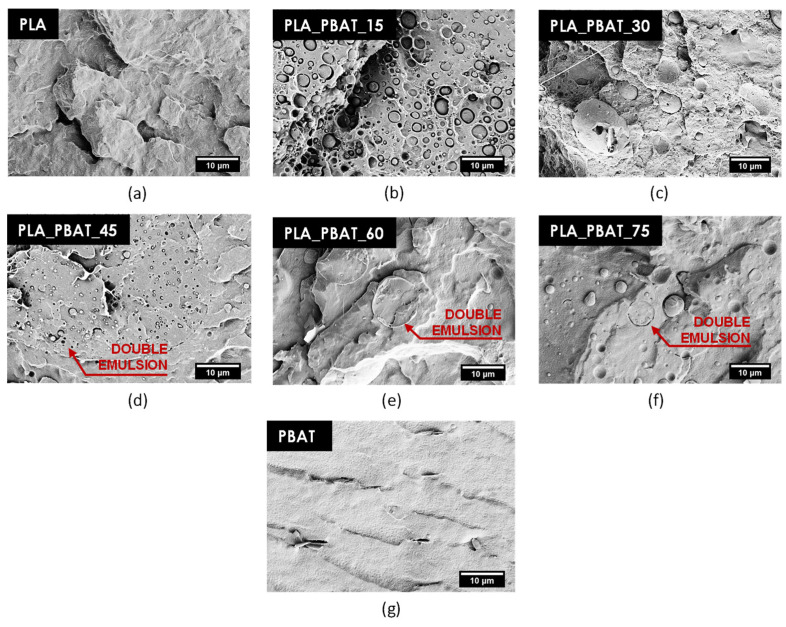
SEM micrographs of the cryofracture surface of neat PLA, neat PBAT and the prepared blends: (**a**) PLA; (**b**) PLA_PBAT_15; (**c**) PLA_PBAT_30; (**d**) PLA_PBAT_45; (**e**) PLA_PBAT_60; (**f**) PLA_PBAT_75; and (**g**) PBAT.

SEM observations were also performed on these blends in order to obtain further information about the interfacial adhesion conditions. The micrographs of the cryofracture surface of the prepared blends are presented in Figure 4a–g.

Neat PLA and PBAT (Figure 4a,g) present a different cryofracture surface appearance, which helps in identifying the two components in the blends (Figure 4b–f). In particular, the cryofracture surface of PLA appears rough (Figure 4a), while that of PBAT is smoother (Figure 4g). The micrographs of the blends reported in Figure 4b–f show that the interfacial adhesion between PLA and PBAT is rather poor, since voids are clearly visible at the interface. The neat separation between the two phases and the presence of detachment phenomena, mostly visible in the PLA_PBAT_15 and PLA_PBAT_45 samples (Figure 4b,d), are evidence of the immiscibility and the poor compatibility of these two polymers. In future works, the interfacial adhesion needs to be improved by using suitable compatibilizers.

The SEM images reveal the presence of very small domains in the order of 1–5 µm (of PLA in the PLA_PBAT_15 and PLA_PBAT_30 samples, and of PBAT in the PLA_PBAT_60 and PLA_PBAT_75 samples) dispersed in the major phase, which has not been possible to observe in the optical micrographs. This confirms the broad domain size distribution already detected in Figure 3a,b,d,e. Furthermore, as indicated by the red arrows in Figure 4d–g, double-emulsion structures can be detected at this magnification level.

### 3.3. Thermal Properties

**Figure 5:**
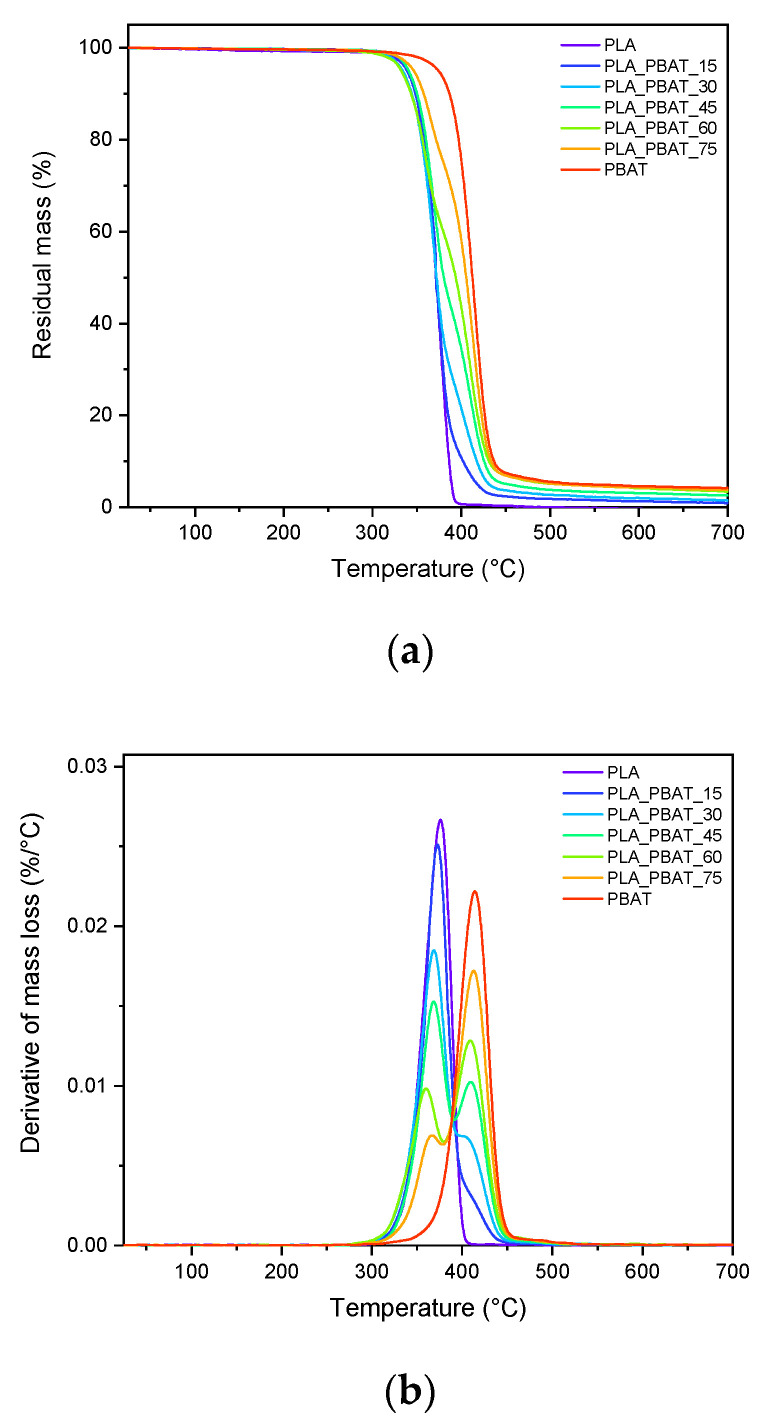
TGA thermograms of neat PLA, neat PBAT and their blends. Trends of (**a**) residual mass and (**b**) mass loss derivative as a function of temperature.

Thermogravimetric tests were performed in order to investigate the thermal degradation behavior of the prepared blends and to explore the influence of blend composition on their degradation resistance. The thermogravimetric curves of the prepared samples along with the corresponding derivative curves are presented in Figure 5a,b, while the most significant results are reported in Table 2.

As it is possible to observe from Figure 5a,b, the thermal degradation of neat polymers occurs in one single step. PLA and PBAT start degrading at temperatures close to 340 °C and 370 °C, respectively, and show maximum degradation rates at 376 and 414 °C, as reported in Table 2. The higher degradation resistance of PBAT is probably attributable to the presence of benzene rings in its molecular structure [56]. A plateau in the TGA curves above 400 °C for PLA and above 450 °C for PBAT can be noticed, highlighting that no significant mass loss in the nitrogen atmosphere occurs at higher temperatures. As for the residue at 700 °C (m_700_), PLA completely degrades without leaving any solid residue, while PBAT presents an m_700_ value of 4.2%. This indicates that partial coking and carbonization occurs for PBAT. Consequently, it is possible to observe that the residual mass increases proportionally to the PBAT amount in the samples.

The decomposition process of all the PLA/PBAT blends takes place in two different steps, associated with the thermal degradation of the PLA and PBAT phases. This is further evidence of the immiscibility of these polymers. With the increasing PBAT concentration, the thermal stability of the blends slightly increases. Indeed, as can be seen from Table 2, the temperature corresponding to a 5% weight loss (T_5%_) shifts to higher temperatures, moving from the PLA_PBAT_15 to PLA_PBAT_75 sample.

**Figure 6:**
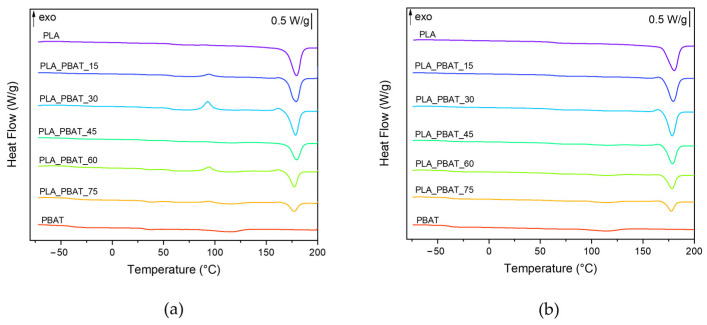
DSC thermograms of neat PLA, neat PBAT and PLA/PBAT blends. (**a**) First heating scan, (**b**) second heating scan.

In order to investigate the thermal properties of the PLA/PBAT blends, a DSC analysis has been carried out. The DSC thermograms collected in the first and second heating scans of the neat PLA, neat PBAT and PLA/PBAT blends are shown in Figure 6a,b, while the most significant results are reported in Table 3. The DSC tests collected in the cooling scan did not provide noteworthy results and were therefore not reported for the sake of brevity.

By analyzing the results reported in Table 3 and in the DSC thermograms in Figure 6a,b, it is possible to observe that the DSC traces of neat PLA and PBAT present the typical profile of semi-crystalline materials. In the first heating scan, the melting point of neat PLA is 178.0 °C, while the glass transition temperature is 57.6 °C. The crystallinity degree of neat PLA is very high (62.1%) and no cold crystallization peaks are observable, probably because the preliminary drying promoted the crystallization of the material. As it is possible to observe from Figure 6a, the endothermic peak of PLA is preceded by a small exothermic peak. According to the literature, this signal corresponds to the reorganization of the α’-crystal of PLA into α-crystals [57]. The α-phase is an ordered structure produced at a high crystallization temperature, while the α’-phase is a metastable disordered phase that develops at low temperatures. Above 150 °C, the α’-crystals become unstable and the phase transformation into α-crystals takes place. In regards to PBAT, it shows a T_g_ at −39.7 °C and a weak and broad endothermic melting peak at 113.7 °C, indicating its low crystallization rate and limited crystallinity degree (10.4%).

Focusing on the first heating DSC scan of the PLA/PBAT blends, it is interesting to observe that the T_m_ and T_g_ values of the PLA and PBAT phases in the blends correspond to those detected for neat polymers, and are thus unaltered by the addition of another phase in the blends. This experimental evidence, together with the well-distinct melting phenomena of the two constituents, confirms that the PLA and PBAT phases are immiscible. In the PLA_PBAT_15 sample, the weak signals associated with the glass transition and melting of the PBAT phase do not permit the determination of the T_g_ and T_m_ for this polymer. In all the blends except for PLA_PBAT_45, an exothermic peak at a temperature close to 93 °C is noted, attributable to the cold-crystallization of PLA macromolecules, which, after the glass transition, acquire sufficient energy to rearrange and crystallize. The crystallinity degree of PLA is significantly reduced in the blends, especially in the PLA_PBAT_15 and PLA_PBAT_30 samples. This suggests that the PLA crystallization process of PLA is hindered by the presence of a second phase in the blend. On the other hand, the peculiar morphology of the PLA_PBAT_45 blend, characterized by the presence of large PBAT domains, has probably favored the crystallization of PLA. Indeed, the crystallization degree of this sample is 62.7%, comparable of that of neat PLA. The crystallization rate of PBAT is also impaired by the presence of PLA in the blends, since the χ_PBAT_ in the blends is lower than that of the neat PBAT.

In the second heating scan, the cold crystallization peak of PLA disappears. This confirms that water cooling after hot pressing partially hindered the crystallization of PLA within the blends. In comparison to the first heating stage, the crystallization degree of both PLA and PBAT is higher in almost all the samples, close to the values of the neat constituents. Finally, no significant differences in the T_m_ and T_g_ of PLA and PBAT determined by the first and second heating scan can be detected.

### 3.4. Mechanical Properties

**Figure 7:**
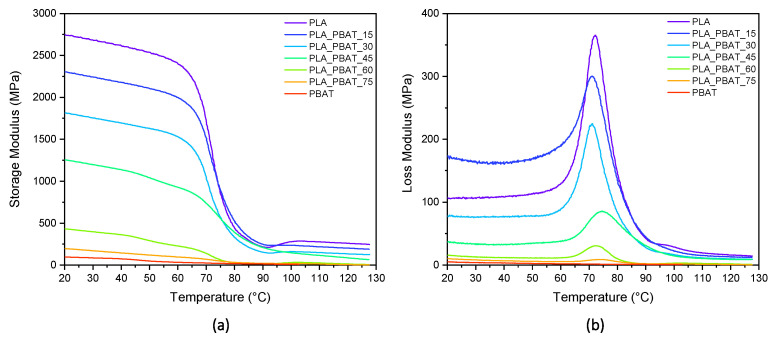
Storage modulus (**a**) and loss modulus (**b**) of neat PLA, neat PBAT and PLA/PBAT blends.

This work, as explained above, aims to evaluate the shape memory performance of PLA/PBAT blends using the glass transition of PLA as the switching temperature. In order to identify the temperature range in which the shape memory behavior of the prepared materials should be evaluated, a DMA analysis was carried out. The trends of the storage modulus and loss modulus as a function of temperature are presented in Figure 7a,b, while the results of the DMA tests on the prepared samples are reported in Table 4.

As is visible from Figure 7a,b, the glass transition temperature of PLA covers a temperature range between 55 °C and 85 °C.. The intensity of the loss modulus peak, associated with this transition, progressively decreases and broadens with the increasing PBAT concentration in the blends, while the corresponding peak remains for all the samples close to a temperature of 70–80 °C. The storage modulus of the blends decreases significantly in the range of 65–80 °C and the drop becomes more pronounced with the increasing PLA content in the samples. Above 90 °C, the cold crystallization of PLA occurs and the modulus rises because of the presence of newly formed crystals. This is particularly evident for the PLA, PLA_PBAT_15, PLA_PBAT_30 and PLA_PBAT_60 samples. It is important to underline that the DSC thermograms of the neat PLA do not reveal any cold crystallization peaks. It can be hypothesized that the lower heating rate used and the orientation of the PLA macromolecules in the stress direction obtained in the DMA tests have probably promoted the cold crystallization of the neat PLA.

Since at 25 °C and at 85 °C the PLA phases in the blends are, respectively, in the glassy and rubbery states, these temperatures were selected for exploring the shape memory performance of the samples. More specifically, 85 °C was chosen as the temperature at which the samples were deformed to the temporary shape. On the other hand, 25 °C was chosen as the temperature at which the deformed samples were cooled down to fix the temporary shape. To avoid any interference with the cold crystallization phenomenon of PLA, temperatures higher than 85 °C were not considered to explore the shape memory behavior. The storage moduli of the samples at 25 °C and at 85 °C are reported in Table 4. By passing from 25 to 85 °C, all the blends experience a storage modulus drop of about one order of magnitude. However, it is possible to notice that the higher the PLA content in the sample, the higher the E’ difference at the two considered temperatures. Since the greater the drop in the storage modulus, the better the shape memory performances of the material, higher shape memory effects are expected in blends with low PBAT contents.

**Figure 8:**
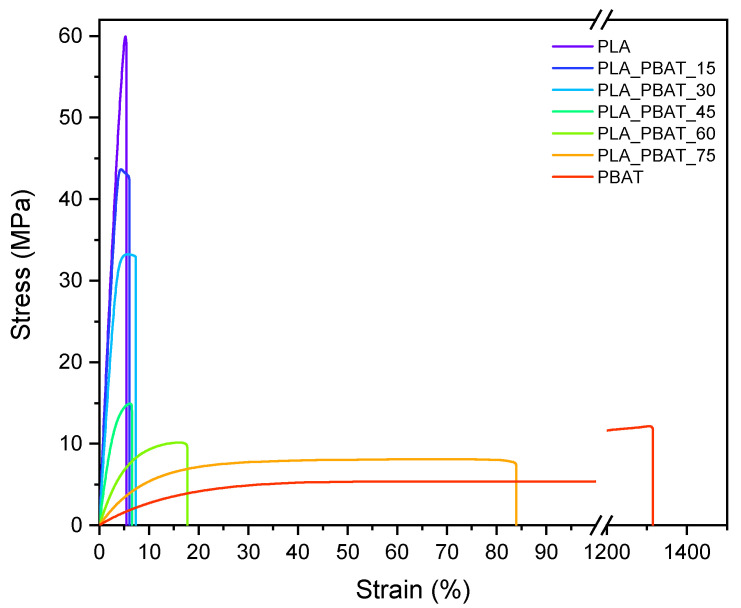
Representative stress–strain curves of neat PLA, neat PBAT and PLA/PBAT blends.

Quasi-static tensile tests were carried out in order to investigate the influence of the PLA/PBAT relative ratio on the mechanical properties of the blends. The representative stress–strain curves of the neat PLA, neat PBAT and of PLA/PBAT blends are shown in Figure 8, while the main results are reported in Table 5.

Neat PLA is characterized by an average tensile strength of 59.2 MPa, a Young’s modulus of 4119.4 MPa and a strain at break of 5.8%. On the other hand, neat PBAT presents a much lower tensile strength (10.7 MPa) and elastic modulus (87.7 MPa), but a significantly greater strain at break (1237.8%). As can be seen from Table 5, the addition of PBAT to PLA leads to a general decrease in stiffness and strength, while the elongation at break remains almost comparable to that of PLA up to a PBAT concentration of 45 wt%. This means that as long as PLA is the matrix constituent of the blend, negligible toughening effects can be obtained, probably due to the low interfacial adhesion between the two phases and the intrinsic, relative brittleness of PLA. The limited interfacial adhesion also explains the considerable σ_b_ drop with limited PBAT contents. A significant increase in blend ductility is noticed for PBAT contents higher than 60 wt%, i.e., when phase inversion has occurred and PBAT becomes the major constituent. In particular, PLA_PBAT_60 and PLA_PBAT_75 are characterized by a strain at break 3.5 and 14.4 times higher than that of neat PLA, respectively. However, it is interesting to note that, even though PBAT constitutes the matrix of these blends, ε_b_ is decisively lower than that of neat PBAT. The presence of large PLA domains and, once again, the low adhesion between the two phases are probably responsible for this result. Regarding the elastic modulus and tensile strength, the prepared blends exhibit high values of E and σ_b_ up to a PBAT content of 30 wt%, while experiencing a drop for higher PBAT concentrations. Once more, this can be explained by recalling the blend morphology. As it was observed in Figure 3a,b, in the PLA_PBAT_15 and PLA_PBAT_30 samples, the PBAT phase is evenly dispersed in the PLA matrix, and the dimensions of the domains are smaller compared to those of the other samples. Moving to the PLA_PBAT_30 to PLA_PBAT_45 sample, the morphology completely changes. Large and irregularly shaped PBAT domains appear, and the interfacial area increases, causing a reduction in blend stiffness and strength. In the future, it will be interesting to enhance the interfacial adhesion between the two phases by using suitable compatibilizers. In conclusion, it is possible to say that when PBAT is added to the PLA matrix as a dispersed component, the tensile strength and elastic modulus decrease but the elongation at break remains the same. In contrast, when PBAT is the major constituent, i.e., PLA is the dispersed component, the stiffness and strength increase with the increasing PLA content, but the elongation at break is significantly reduced.

The elastic modulus of the blends was theoretically predicted by using three models, i.e., the Series, Parallel and Equivalent Box models. More specifically, the Series and Parallel models were used to predict the upper and lower boundaries of blend stiffness [58], according to the expressions reported in Equations (4) and (5), respectively.

(4) $$E_{b\;}=E_{PLA}\cdot V_{PLA}+E_{PBAT}\cdot V_{PBAT}$$

(5) $$E_{b}=\frac{E_{PLA}\cdot E_{PBAT}}{\left(V_{PLA}\cdot E_{PBAT}+V_{PBAT}\cdot E_{PLA}\right)}$$

where *E_b_* is the elastic modulus of the blend, E_PLA_ and E_PBAT_ are the elastic moduli of neat PLA and PBAT, respectively, while V_PLA_ and V_PBAT_ are the volume fractions of PLA and PBAT in the samples. The Parallel model assumes that the continuous phase consists of the higher modulus polymer and therefore represents the upper boundary. On the other hand, the lower boundary is represented by the Series model, which assumes that the lower modulus component is the continuous phase. The Equivalent Box model (EBM) combined with the phase continuity percolation approach was also used to predict the modulus of these polymer blends [59]. The EBM operates with partly parallel (subscript p) and partly series (subscript s) couplings of two components. It is a two-parameter model in that of the four volume fractions, only two are independent variables. The volume fractions of the constituents are correlated as reported in Equations (6) and (7):

(6) $$V_{PLA\;}=(V_{PLAp}+V_{PLAs})$$

(7) $$V_{PBAT\;}=(V_{PBATp}+V_{PBATs})$$

where V_PLA_ + V_PBAT_ = V_PLAs_ + V_PLAp_ + V_PBATs_ + V_PBATp_ = 1. The tensile modulus of the blend (E_b_) is given as reported in Equation (8):

(8) $$E_{b\;}=E_{p}\cdot V_{p}+E_{s}\cdot V_{s}=E_{PLA}\cdot V_{PLAp}+E_{PBAT}\cdot V_{PBATp}+\frac{V_{s}^{2}}{\frac{V_{PLAs}}{E_{PLA}}+\frac{V_{PBATs}}{E_{PBAT}}}$$

where E_p_ and E_s_ are, respectively, the elastic moduli of the parallel and series branch of the system, V_p_ is the sum of V_PLAp_ and V_PBATp_, and V_s_ is the sum of V_PLAs_ and V_PBATs_. Using the universal formula provided by percolation theory for the elastic modulus of binary systems, Kolarik and coworkers derived equations for the determination of the volume fractions in parallel, as reported in Equations (9) and (10):

(9) $$V_{PLAp\;}={\left(\frac{V_{PLA}-V_{PLAcr}}{1-V_{PLAcr}}\right)}^{T}$$

(10) $$V_{PBATp\;}={\left(\frac{V_{PBAT}-V_{PBATcr}}{1-V_{PBATcr}}\right)}^{T}$$

where V_PLAcr_ and V_PBATcr_ are the critical volume fractions at which the component PLA and PBAT become partially continuous and T is the critical exponent. The best fit of the experimental results was obtained with V_PLAcr_ = V_PBATcr_ = 0.1 and T = 1.6. For further details about the EBM, the reader can refer to the work of Kolarik et al. [59].

**Figure 9:**
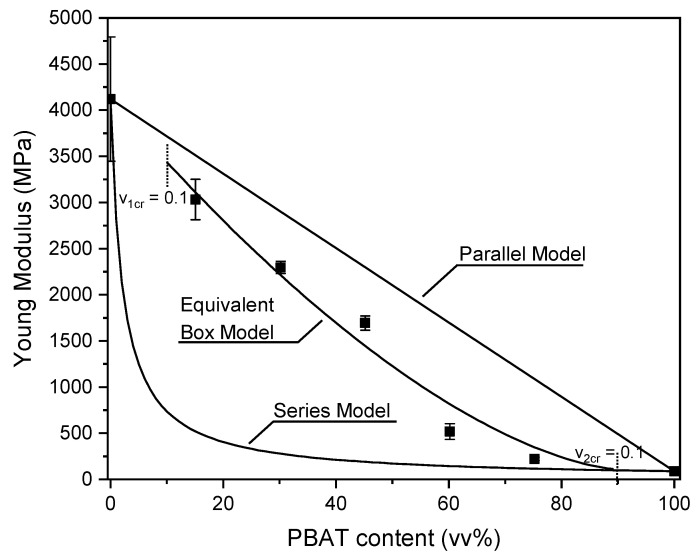
Experimental and theoretical values of the elastic modulus of the produced blends as a function of the PBAT content.

The experimental and theoretical values of the elastic modulus of the prepared blends as a function of the volume fraction of PBAT are shown in Figure 9.

All the elastic modulus values fall into the range between the Parallel and Series models, suggesting that PLA and PBAT are partially compatible, even though they are not miscible. It is interesting to notice that the experimental results are in good agreement with the theoretical predictions based on the Equivalent Box model. Finally, the elastic modulus of the PLA_PBAT_75 sample predicted by the Series model practically coincides with the experimental value. This indicates that for this composition, PBAT is the continuous phase with PLA dispersed within it.

### 3.5. Evaluation of the Shape Memory Behavior

Testing the shape memory properties of polymers using a DMA apparatus in single cantilever mode is quite useful when one needs to compare the shape memory performance of materials showing different elongation at break values. Table 6 shows the strain fixity and strain recovery parameters of the neat PLA, neat PBAT and PLA/PBAT blends.

As expected, neat PLA is characterized by a good strain fixity capability, reaching an SF value of 83.8%. It is worth noting that the strain recovery of neat PLA is quite limited, considering the values reported in the literature [10,11,14,15,60,61,62,63,64,65,66]. Only slightly more than half of the imposed deformation (56.7%) is recovered during the recovery step. This experimental result can probably be attributed to its high crystallinity degree. However, to confirm this hypothesis, future works will need to focus on varying the processing conditions to obtain a PLA with lower crystallinity and investigating its shape memory properties. In regards to neat PBAT, an excellent strain fixability (SF value of 91.3%) and negligible recovery capabilities (SR value of 8.7%) have been found. Due to the absence of a transition temperature in the explored temperature range, neat PBAT cannot exhibit a shape memory behavior.

**Figure 10:**
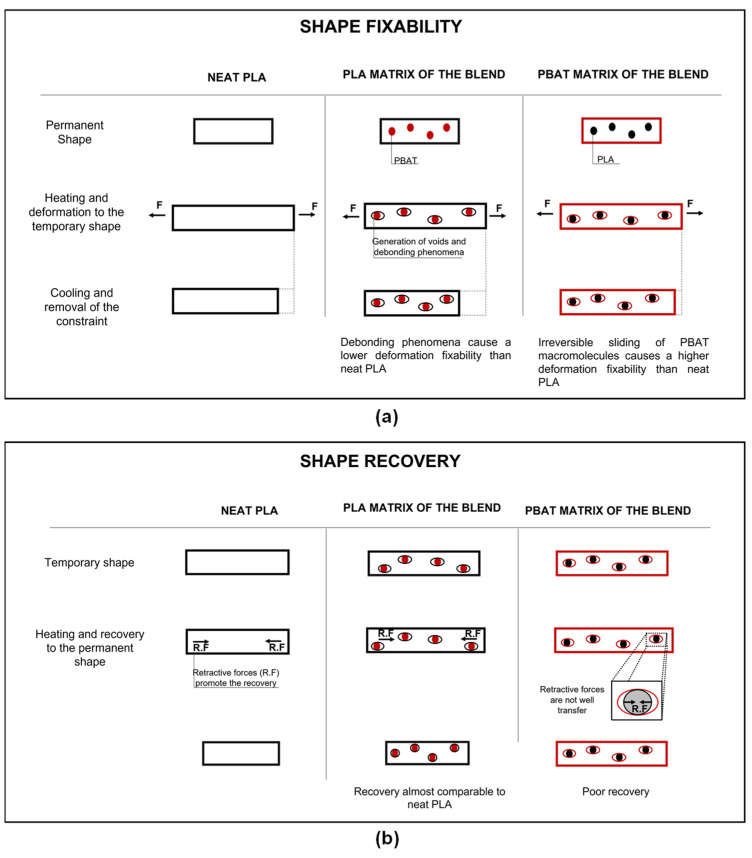
Schematization of the proposed mechanism for the shape memory behavior of the produced blends. Model of (**a**) strain fixity and (**b**) strain recovery behavior.

With the addition of PBAT, the shape memory performance of PLA undergoes a particular trend. As can be seen from Table 6, while the fixing capability tends to slightly decrease up to a PBAT concentration of 45% and then to increase again for higher PBAT amounts, the strain recovery parameter exhibits almost the opposite trend. It increases up to a PBAT content of 30 wt% and then decreases. The higher SR parameters of PLA_PBAT_15 and PLA_PBAT_30 compared to neat PLA could be probably related to the lower crystallinity degree of PLA in these blends, as evidenced by the DSC analyses. The samples constituted by a PBAT matrix (PLA_PBAT_60 and PLA_PBAT_75) fix the deformation well but show inferior recovery capabilities. In particular, in the PLA_PBAT_75 blend, the SR is almost complementary to the SF parameter, indicating that just a very small deformation is recovered during the recovery step. The higher fixability and the lower recovery capabilities of these blends are probably attributable to two aspects, as illustrated in Figure 10a,b. The first is related to the fact that PBAT is a flexible thermoplastic polymer characterized by very low crystallinity. The crystalline domains should act as net points, avoiding the irreversible sliding of the macromolecules when the polymer is mechanically stressed. However, since the crystallinity degree of PBAT is very low, part of the polymer chains slides irreversibly and, when the stress is released, a great deal of the deformation imposed is maintained. The second aspect is related to the poor adhesion between the PLA and PBAT phases, which probably causes a non-optimal stress transfer between the two constituents. PLA provides at the same time both the transition temperature that guarantees the fixability of the temporary shape and the retractive forces that allow the material to recover the original permanent shape. In more detail, the net points, or the crystalline domains, determine the permanent shape of the polymer. The glassy area, instead, acts as a molecular switch and is responsible for the fixability of the temporary shape. Consequently, the PLA domains should act as a spring in the PBAT matrix and guarantee recovery to the initial condition. However, the poor adhesion between the phases does not allow a good transfer of these retractive forces from the PLA domains to the PBAT matrix and, as a result, the blend exhibits poor recovery capabilities (Figure 10b). To confirm this hypothesis, it will be useful in future works to perform the same shape memory tests on compatibilized blends. Furthermore, the difference in SR parameters among PLA_PBAT_60 and PLA_PBAT_75 (54.7% and 14.3%, respectively) is probably due to the morphologies these blends show. Both PLA_PBAT_60 and PLA_PBAT_75 are high-PBAT-content blends; however, while PLA_PBAT_60 presents a co-continuous structure, PLA_PBAT_75 exhibits a sea-island morphology (Figure 3d,e). The elongated and interconnected PLA domains in PLA_PBAT_60 probably promote a higher recovery capability with respect to that of PLA_PBAT_75. It has already been mentioned that only PLA presents a shape memory behavior, and only PLA can provide the retractive forces to allow the blend to return to the original conditions. Consequently, the presence of larger PLA domains in the blend is beneficial from the point of view of the recovery performances.

The best compromise between shape fixability and recovery capability was obtained in the PLA_PBAT_30 sample, for which SF and SR values of 77.3% and 73.6% were obtained, respectively. However, it should be noticed that the compositions exhibiting the more interesting shape memory properties do not coincide with the blends showing a significant improvement in PLA ductility. Once again, the addition of a proper compatibilizer could give a satisfactory answer to this issue.

## 4. Conclusions

The present work investigated the microstructural, thermomechanical and shape memory properties of biodegradable PLA/PBAT blends produced by melt compounding at different relative amounts. Rheological measurements, optical microscopy and SEM micrographs evidenced the immiscibility and the low interfacial adhesion between the PLA and PBAT phases. Depending on the PLA/PBAT ratio, the two constituents exhibited either a sea-island morphology or an almost co-continuous structure, and it was shown that phase inversion occurred in a PBAT concentration interval between 45 wt% and 60 wt%. The immiscibility of these blends was also confirmed by a DSC analysis, as the glass transition temperatures of PLA and PBAT corresponded to the values of neat polymers and were unaffected by the blend composition. The TGA analysis revealed that the addition of PBAT slightly improved the thermal stability of PLA, probably because of the presence of rigid benzene rings in the molecular structure of PBAT.

Tensile tests evidenced that the stiffness and strength of the blends decreased with the increasing PBAT concentration, while the elongation at break remained comparable to that of neat PLA (5.8%) up to a PBAT content of 45 wt%. A significant increment in blend ductility was registered only for higher PBAT concentrations (elongation at break up to 83.8% with a PBAT amount of 75 wt%).

The shape memory performance of PLA was impaired by the addition of PBAT. In particular, a reduction in the strain recovery parameter was registered with the increasing PBAT content, probably due to the low adhesion between the constituents. The best compromise between shape fixability and recovery capability was obtained with a PBAT content of 30 wt% and SF and SR values of 77.3% and 73.6%, respectively.

These results constitute a basis for future research on innovative multifunctional biodegradable polymer blends with promising applications in the packaging field and suggest that their properties could be enhanced by applying suitable compatibilizers. Future works should focus on the selection of the most appropriate compatibilizer, the effect of blend compatibilization on the physical and shape memory properties of these systems and a deep investigation of the gas permeability of the blends.

## Figures and Tables

**Table 1:** List of the prepared samples and their nominal composition.

Sample	PLA Content (wt%/vv%)	PBAT Content (wt%/vv%)
PLA	100.0/100.0	-
PLA_PBAT_15	85.0/84.9	15.0/15.1
PLA_PBAT_30	70.0/69.8	30.0/30.2
PLA_PBAT_45	55.0/54.8	45.0/45.2
PLA_PBAT_60	40.0/39.8	60.0/60.2
PLA_PBAT_75	25.0/24.8	75.0/75.2
PBAT	-	100.0/100.0

**Table 2:** Results of the TGA tests on neat PLA, neat PBAT and their blends.

Sample	T_5%_ (°C)	T_peak1_ (°C)	T_peak2_ (°C)	m_700_ (%)
**PLA**	339.7	376.1	-	0.0
**PLA_PBAT_15**	341.2	372.6	-	0.9
**PLA_PBAT_30**	341.8	368.8	406.0	1.5
**PLA_PBAT_45**	343.7	368.5	409.9	2.6
**PLA_PBAT_60**	345.3	360.0	409.4	3.4
**PLA_PBAT_75**	346.5	366.4	412.6	3.8
**PBAT**	374.3	-	414.4	4.2

T_5%_ = temperature corresponding to a mass loss of 5%; T_peak1_ = temperature of the maximum degradation rate of PLA; T_peak2_ = temperature of the maximum degradation rate of PBAT; m_700_ = mass residue at 700 °C.

**Table 3:** Results of the DSC tests on neat PLA, neat PBAT and PLA/PBAT blends (first and second heating scans).

Sample	T_gPBAT_ (°C)	T_gPLA_ (°C)	T_ccPLA_ (°C)	ΔH_ccPLA_ (J/g)	T_mPBAT_ (°C)	ΔH_mPBAT_ (J/g)	T_mPLA_ (°C)	ΔH_mPLA_ (J/g)	χ_PLA_ (%)	χ_PBAT_ (%)
1st heating										
PLA	-	57.6	-		-		178.0	58.3	62.2	-
PLA_PBAT_15		58.9	93.3	20.3				48.6	35.8	
PLA_PBAT_30	−36.9	56.5	93.0	15.2			177.1	39.8	37.7	
PLA_PBAT_45	−39.1	57.3	-	-	114.1	2.7	178.5	32.1	62.7	6.3
PLA_PBAT_60	−39.6	56.5	93.8	6.9	115.2	3.3	176.5	22.2	41.2	4.9
PLA_PBAT_75	−39.1	58.5	93.7	1.8	114.7	5.9	176.1	13.0	47.9	6.9
PBAT	−39.7	-	-	-	113.7	11.9	-	-	-	10.4
2nd heating										
PLA	-	62.4	-	-	-	-	179.1	54.5	58.6	-
PLA_PBAT_15	−44.1	61.4	-	-			178.0	43.0	54.4	
PLA_PBAT_30	−41.9	61.2	-	-			177.3	38.6	59.3	
PLA_PBAT_45	−39.5	59.2	-	-	114.0	3.2	177.6	31.4	61.5	6.3
PLA_PBAT_60	−39.9	59.0	-	-	114.0	4.7	177.4	22.1	59.4	6.9
PLA_PBAT_75	−38.5	59.1	-	-	113.4	6.3	176.5	14.0	56.6	7.5
PBAT	−39.0	-	-	-	114.2	11.2	-	-	-	9.8

T_gPBAT_ = glass transition temperature of PBAT; T_gPLA_ = glass transition temperature of PLA; T_ccPLA_ = cold crystallization temperature of PLA; T_mPBAT_: melting temperature of PBAT; ΔH_mPBAT_ = melting enthalpy of PBAT; T_mPLA_: melting temperature of PLA; ΔH_mPLA_ = melting enthalpy of PLA; *χ*_PLA_= crystallinity degree of PLA; *χ*_PBAT_= crystallinity degree of PBAT.

**Table 4:** Results of the DMA tests on the prepared blends.

Sample	Storage Modulus at 25 °C (MPa)	Storage Modulus at 85 °C (MPa)
**PLA**	2725.0	278.1
**PLA_PBAT_15**	2279.3	336.5
**PLA_PBAT_30**	1775.0	208.4
**PLA_PBAT_45**	1221.6	276.9
**PLA_PBAT_60**	420.2	19.5
**PLA_PBAT_75**	188.2	24.5
**PBAT**	87.9	15.3

**Table 5:** Result of quasi-static tensile test at 25 °C on the prepared samples.

Sample	E (MPa)	σ_b_ (MPa)	ε_b_ (%)
**PLA**	4119.4 ± 673.3	59.2 ± 2.4	5.8 ± 0.7
**PLA_PBAT_15**	3030.9 ± 219.2	42.2 ± 0.7	6.0 ± 0.3
**PLA_PBAT_30**	2295.4 ± 66.2	32.3 ± 1.1	7.9 ± 1.3
**PLA_PBAT_45**	1694.3 ± 78.2	11.8 ± 3.4	5.5 ± 1.4
**PLA_PBAT_60**	221.9 ± 17.9	9.2 ± 0.3	20.3 ± 2.9
**PLA_PBAT_75**	87.7 ± 17.9	7.7 ± 0.6	83.8 ± 12.2
**PBAT**	87.7 ± 2.0	10.7 ± 1.2	1237.8 ± 136.1

E= elastic modulus; σ_b_= stress at break; ε_b_= strain at break.

**Table 6:** Strain fixity and strain recovery parameters of neat PLA, neat PBAT and PLA/PBAT blends.

Sample	SF (%)	SR (%)
**PLA**	83.8	56.9
**PLA_PBAT_15**	81.9	63.5
**PLA_PBAT_30**	77.3	73.6
**PLA_PBAT_45**	73.1	64.3
**PLA_PBAT_60**	80.2	54.7
**PLA_PBAT_75**	91.4	14.3
**PBAT**	91.3	8.7

## Data Availability

Data available on request.

## Supplementary Materials

The following supporting information can be downloaded at: https://www.mdpi.com/article/10.3390/polym15040881/s1, Figure S1: Thermo-mechanical cycle of (a) PLA_PBAT_15, (b) PLA_PBAT_30, (c) PLA_PBAT_45, (d) PLA_PBAT_60, (e) PLA_PBAT_75.
