# Supplementary figures and images for: Evaluation of the Physical and Shape Memory Properties of Fully Biodegradable Poly(lactic acid) (PLA)/Poly(butylene adipate terephthalate) (PBAT) Blends
Source: Polymers (Basel). 2023 Feb 10;15(4):881. doi: 10.3390/polym15040881 (PMC9963890; doi:10.3390/polym15040881)

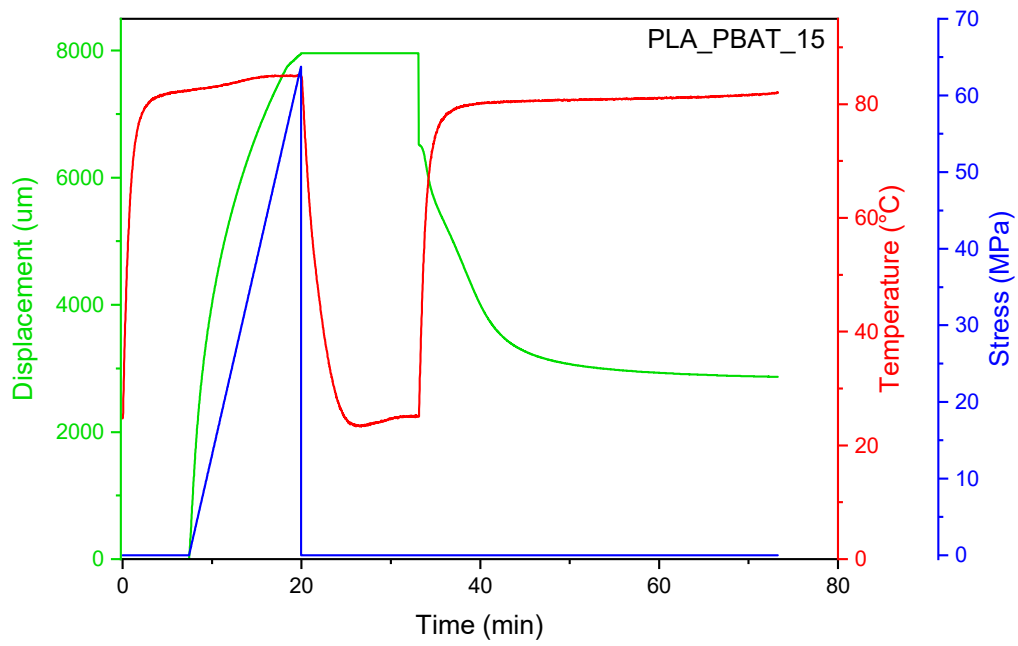

(a)

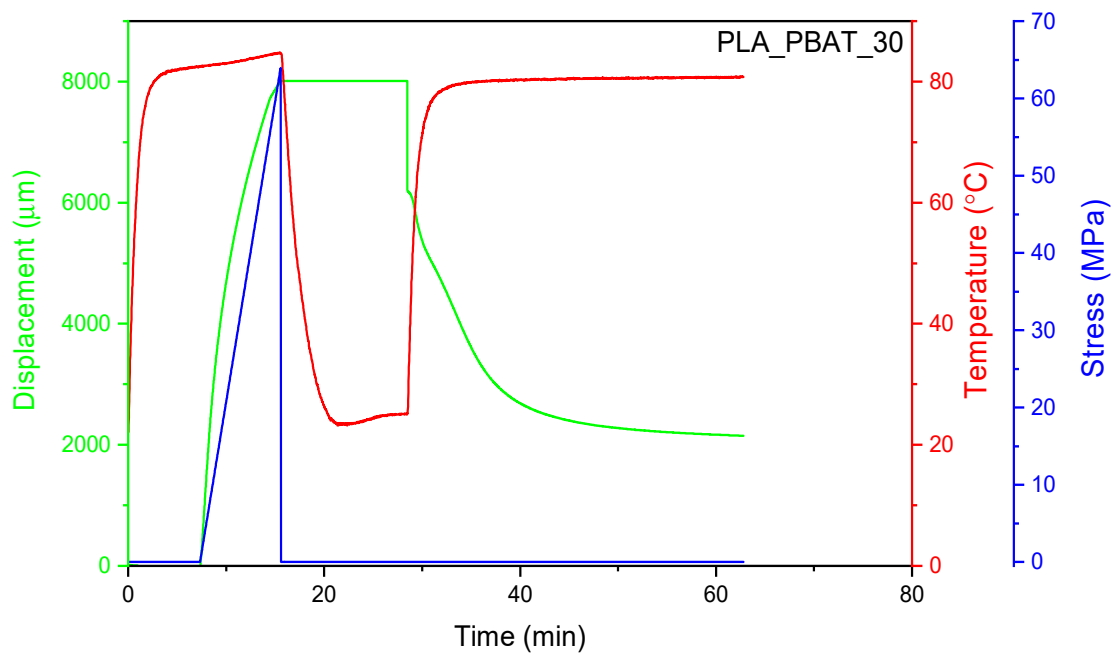

(b)

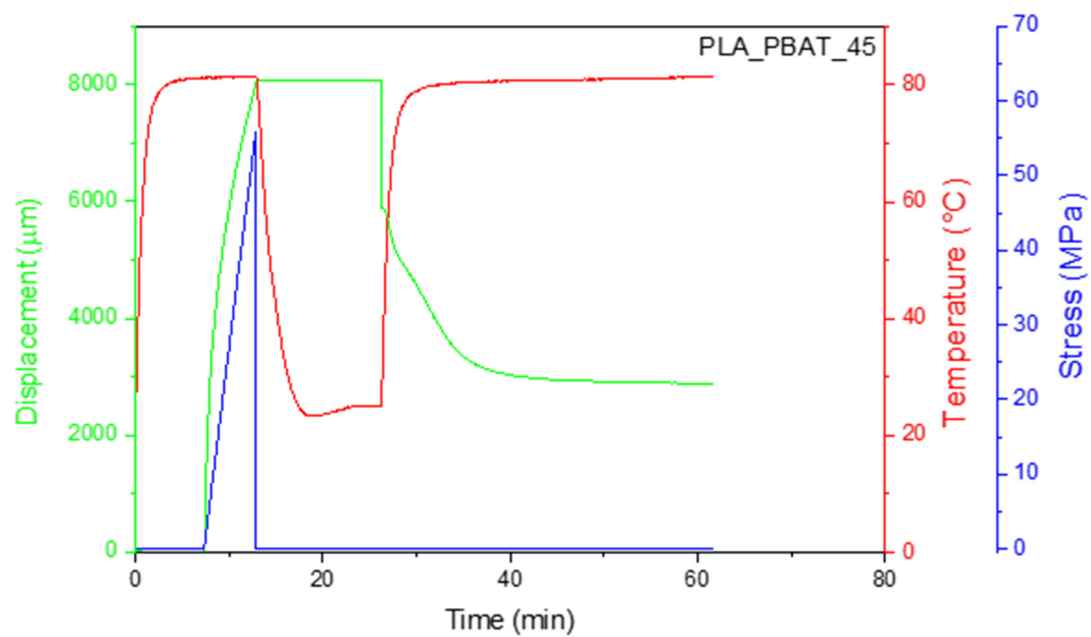

(c)

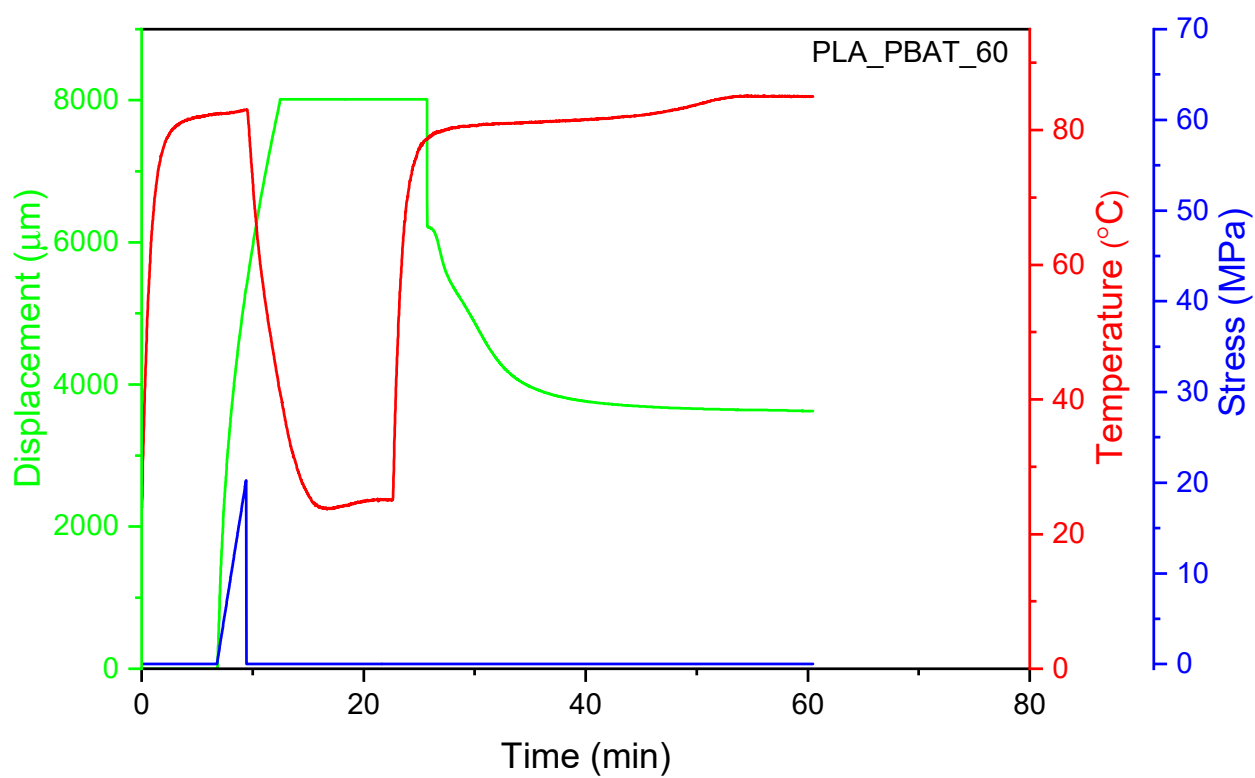

(d)

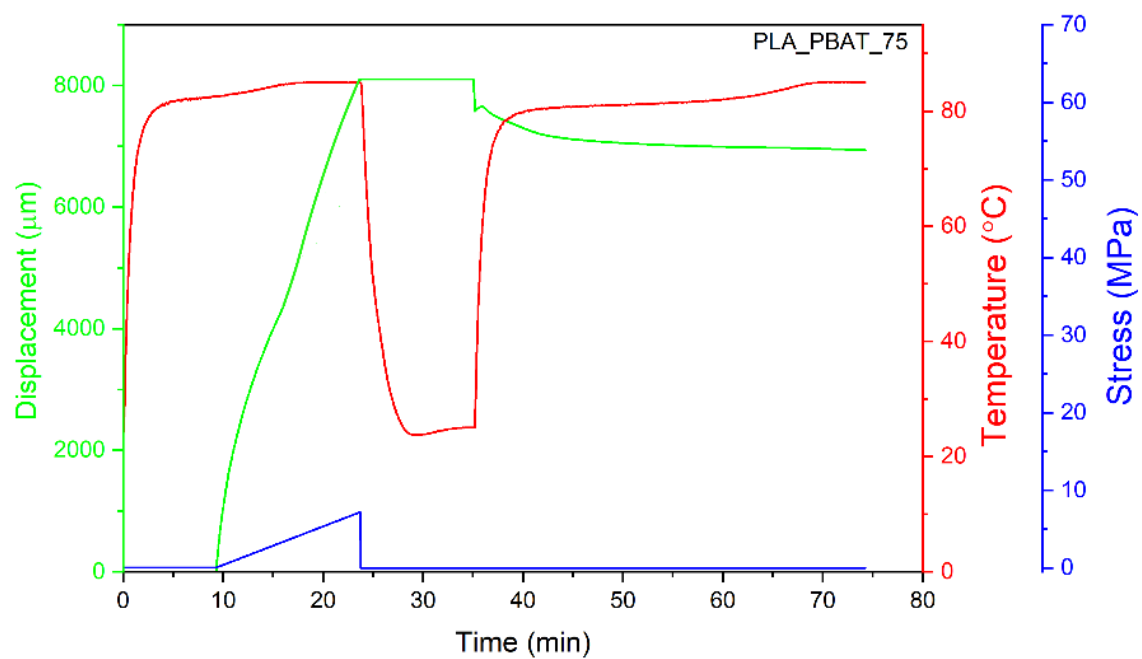

(e)

**Figure S1.** Thermo-mechanical cycle of (a) PLA\_PBAT\_15, (b) PLA\_PBAT\_30, (c) PLA\_PBAT\_45, (d) PLA\_PBAT\_60, (e) PLA\_PBAT\_75.

Supplement: Supplementary file 1 [file polymers-15-00881-s001.zip › polymers-2139587-supplementary.pdf]
